# Complete Genome Sequence of *Paenibacillus* sp. Strain VT 400, Isolated from the Saliva of a Child with Acute Lymphoblastic Leukemia

**DOI:** 10.1128/genomeA.00894-15

**Published:** 2015-08-13

**Authors:** George Tetz, Victor Tetz, Maria Vecherkovskaya

**Affiliations:** a Institute of Human Microbiology, LLC, New York, New York, USA; b First State I. P. Pavlov Medical University, Saint Petersburg, Russian Federation

## Abstract

We report here the complete genome sequence of spore-forming *Paenibacillus* sp. strain VT 400, isolated from the saliva of a child with acute lymphoblastic leukemia. The genome consists of 6,986,122 bp, with a G+C content of 45.8%. It possesses 5,777 predicted protein-coding genes encoding multidrug resistance transporters, virulence factors, and resistance to chemotherapeutic drugs.

## GENOME ANNOUNCEMENT

The genus *Paenibacillus* comprises aerobic or facultatively anaerobic, rod-shaped, immotile or motile (via peritrichous flagella), endospore-forming bacteria. Species of this genus have been isolated from soil, water, plants, milk, and other media (1–3). *Paenibacillus* spp. were not known to cause human disease until recent reports showed the possible involvement of *P. alvei, P. thiaminolyticus*, and *P. sputi* in human diseases (4–6).

*Paenibacillus* sp. strain VT 400 was isolated from the saliva of a pediatric patient with acute lymphoblastic leukemia. Complete sequencing of its 16S rRNA gene showed 99% similarity with that of *Paenibacillus amylolyticus*, a pectinase producer found in the larval hindgut of the fly. To date, *P. amylolyticus* has not been detected in humans. Biochemical characterization and matrix-assisted laser desorption ionization–time of flight (MALDI-TOF) mass spectrometry have revealed differences between *Paenibacillus* sp. strain VT 400 and other *P. amylolyticus* strains.

Whole-genome sequencing was performed using the Illumina HiSeq 2500 sequencer, according to the manufacturer’s instructions (Illumina GA IIx; Illumina, CA). *De novo* assembly was performed with the SPAdes genome assembler software, version 3.5.0 (7). The assembly resulted in 116 contigs with 125-fold average coverage.

The draft genome of *Paenibacillus* sp. strain VT 400 consists of 6,986,122 bp, with a G+C content of 45.8%. The genome was annotated using the NCBI Prokaryotic Genome Annotation Pipeline (8). It contains 100 tRNA genes, 50 rRNA and two noncoding RNA operons, and 5,777 protein-coding sequences (CDSs). THe analysis revealed genes encoding resistance to chemotherapeutic drugs, like tunicamycin and bleomycin (9, 10). Furthermore, genes of multidrug resistance (MDR) efflux pumps (ABC transporter, a member of the multidrug and toxin extrusion [MATE] family of MDR proteins, and an MDR transporter) and genes encoding resistance to antibiotics, including vancomycin (*vanH*, *vanX*, *vanW*, and *vanZ*), fosmidomycin, tetracycline (*tetA*, *tetB*), as well as β-lactamase genes from superfamilies I to III, were identified. The genome contains genes encoding known virulence factors, like hemolysin D and flagellar and sporulation proteins (11).

In comparison with the genome of *P. amylolyticus* FSL-R5-192 (the phylogenetically closest organism), that of *Paenibacillus* sp. strain VT 400 is smaller (6,986,122 versus 7,083,071 bp) and contains fewer protein-coding genes (5,777 versus 6,163), although the two genomes have a similar G+C content (45.8%). According to DNA-DNA hybridization (DDH) prediction based on genome BLAST distance phylogeny (GBDP), this pair of genomes (*Paenibacillus* sp. strain VT 400 and *P. amylolyticus* FSL-R5-192) has a DDH value of 74.40%, as calculated in the Genome-to-Genome Distance Calculator (GGDC) Web server (GGDC 2.0). This value is below the threshold of 79% for genomes belonging to different subspecies (12, 13).

The most variable regions between two strains were found in the coding regions of transcriptional regulators, RNA polymerase, DNA polymerase, DNA topoisomerase III, endonuclease IV, and spore germination-related genes, which had only 24 to 38% similarity; some genes responsible for antibiotic resistance are absent in *P. amylolyticus* FSL-R5-192.

The complete genome sequence of *Paenibacillus* sp. strain VT 400 will help determine the role of *Paenibacillus* species in human diseases and provide insights into the composition of microbial flora in patients with hematological malignancies.

### Nucleotide sequence accession numbers.

This complete genome sequencing project has been deposited in GenBank under the accession no. LELF00000000. The version described in this paper is the first version, LELF01000000.
